# Association of *ESR1* Mutations and Visceral Metastasis in Patients with Estrogen Receptor-Positive Advanced Breast Cancer from Brazil

**DOI:** 10.1155/2019/1947215

**Published:** 2019-08-14

**Authors:** Tomás Reinert, Guilherme Portela Coelho, Jovana Mandelli, Edinéia Zimermann, Facundo Zaffaroni, José Bines, Carlos Henrique Barrios, Marcia Silveira Graudenz

**Affiliations:** ^1^PPG Ciências Médicas, Universidade Federal do Rio Grande do Sul (UFRGS), Porto Alegre, Brazil; ^2^Centro de Pesquisa da Serra Gaúcha (CEPESG), Caxias do Sul, Brazil; ^3^Latin American Cooperative Oncology Group (LACOG), Porto Alegre, Brazil; ^4^Grupo Diagnose Patologia, Genética e Biologia Molecular, Caxias do Sul, Brazil; ^5^FARO STAT Solutions, Porto Alegre, Brazil; ^6^Instituto Nacional do Câncer (INCA), Rio de Janeiro, Brazil; ^7^Clínica São Vicente, Rio de Janeiro, Brazil

## Abstract

Mutations in the *ESR1* gene (*ESR1m*) are important mechanisms of resistance to endocrine therapy in estrogen receptor-positive advanced breast cancer and have been recognized as a prognostic and predictive biomarker as well as a potential therapeutic target. However, the prevalence of *ESR1m* in real-world patients has not been adequately described. Therefore, we sought to evaluate the prevalence of *ESR1m* in metastatic samples from Brazilian patients with estrogen receptor-positive (ER+) advanced breast cancer previously treated with endocrine therapy. The presence of *ESR1m* was evaluated in formalin-fixed paraffin-embedded (FFPE) breast cancer tissue using real-time quantitative polymerase chain reaction (RT-qPCR). Mutations in codons 380, 537, and 538 of the *ESR1* gene were analyzed. Out of 77 breast cancer samples, 11 (14.3%) showed mutations in the *ESR1* gene. *ESR1m* were detected in a variety of organs, and the D538G substitution was the most common mutation. In visceral metastasis, *ESR1m* were detected in 25% (8/32) of the samples, whereas in nonvisceral metastasis, *ESR1m* were detected in 6.7% (3/45) of the samples. The odds of a sample with visceral metastasis having an *ESR1* mutation is 4.66 times the odds of a sample of nonvisceral metastasis having an *ESR1* mutation (95% CI: 1.13–19.27; *p* value = 0.0333). Our study indicates that the prevalence of *ESR1m* in samples from Brazilian patients with metastatic ER+ breast cancer is similar to that described in patients included in clinical trials. We observed an association of *ESR1m* with visceral metastasis.

## 1. Introduction

Estrogen receptor-positive breast cancer is the most common breast cancer subtype. Endocrine therapy (ET), a targeted treatment to the estrogen receptor (ER) pathway, is the fundamental initial therapeutic approach in all stages of the disease [1]. Nonetheless, clinical resistance associated with progression of disease remains a significant therapeutic challenge [2, 3]. Mutations of the *ESR1* gene, which encodes the ER protein, have been increasingly identified as a mechanism of endocrine resistance [4].

The potential clinical implications of *ESR1* mutations (*ESR1m*) remained underappreciated for more than a decade after its discovery since initial studies focused on primary tumors, where the prevalence of *ESR1m* is very low [5]. Subsequently, it was demonstrated that breast tumors undergo genomic evolution and *ESR1m* have been described in 9–40% of patients with advanced ER+ breast cancer resistance to aromatase inhibitors [3, 4, 6–8]. *ESR1* mutation is a biomarker of worse prognosis and is being evaluated as a predictive biomarker as well as a potential therapeutic target [9].

Despite recent advances in the field, several questions remain unanswered about *ESR1m* such as the prediction of which tumor will develop this mechanism of resistance. At the same time, the majority of data are derived from patients included in clinical trials, more frequently in developed countries, and little is known about mechanisms of ET resistant in real-world patients, especially in the population from low- to middle-income countries. We aimed here to evaluate the prevalence of *ESR1m* in metastatic tumor tissues from breast cancer patients from Brazil.

## 2. Methods

From the archive of the Pathology Department at a single academic center, we collected formalin-fixed paraffin-embedded (FFPE) tissue specimens from consecutive patients enrolled between 2014 and 2017 with recurrent or metastatic breast cancer previously treated with endocrine therapy. Only tumors of ER-positive HER2-negative metachronous metastasis were selected. All hematoxylin and eosin (H&E) and immuno-histochemistry (IHQ) slides from tumor samples were reexamined by a pathologist who confirmed the diagnosis of metastatic carcinoma and quality (amount of reminiscent neoplastic tissue on paraffin-embedded archived tissue) of each specimen. Additionally, all the lesions were diagnosed as breast metastases by IHQ using one or more of the following markers: GATA3, GCDFP-15, and/or mammaglobin.

In each sample, the tumor area was marked by the pathologist and a cut of approximately 35 mg was performed, followed by the extraction of the genetic material (DNA) with the Wizard© Genomic DNA purification kit (Promega). DNA was quantified using Qubit fluorometric quantitation (Thermo Fischer Scientific), and 20 ng/*μ*l was the threshold for the analysis of the mutation. The reactions were performed with the equipment 7500 fast real-time PCR system using TaqMan Genotyping master mix, primers, and TaqMan© probes, from Applied Biosystems (Foster City, CA) following all recommendations of the manufacturer. The analyzed mutations were Y537N, Y537C, Y537S, E380Q, and D538G. To detect the presence of the mutation, a Taqman© reference probe was used, followed by the analysis in the 7500 Software v2.06 (Thermo Fischer Scientific).

A sample size of 81 patients was calculated with an estimated prevalence of 30%, a desired precision of estimate of 0.1 and a confidence level of 0.95. The primary endpoint was the prevalence of *ESR1m*. The secondary endpoint was the association of *ESR1m* and site of metastasis (visceral versus nonvisceral). Data were analyzed using descriptive statistics. Logistic regression was applied in order to estimate the OR (odds ratio) and 95% confidence interval (95% CI). A *p* value less or equal to 0.05 was deemed to be significant. This project was reviewed and approved at the IRB institutional review board (Ethical Committee).

## 3. Results

Seventy-seven samples were included in the analysis. Of the initial 81 selected samples, 4 were removed from the analysis due to an insufficient amount of extracted DNA (all from bone metastasis). The prevalence of ESR mutation was 14.3% (11 samples). *ESR1m* were detected in metastatic tissues from different organs such as pleura (*n* = 3), liver (*n* = 2), lung (*n* = 2), ovary, lymph node, bone, and chest wall. The most frequently detected mutation was the D538G substitution (*n* = 5), followed by mutations in codon 537 (3 Y537N substitutions, 2 Y537C, and 1 Y537S). No mutations in codon 380 were detected. For more information on the molecular biology analysis, see Supplementary Material (available here).

The probability of having an *ESR1* mutation was modeled considering the information regarding local of metastasis (Table 1). In visceral metastasis, *ESR1m* were detected in 25% (8/32) of the samples, whereas in nonvisceral metastasis, an *ESR1m* were detected in 6.7% (3/45) of the samples. Despite the low number of cases with mutation (reflected in the wide CI), the logistic regression showed that the odds of a sample with visceral metastasis having an *ESR1* mutation is 4.66 times the odds of a sample of nonvisceral metastasis having an *ESR1* mutation (95% CI: 1.13–19.27; *p* value = 0.0333).

## 4. Discussion

Estrogen receptor-positive (ER+) tumors are the most frequent form of breast cancer and responsible for most of the deaths caused by this disease [10]. ET is the mainstay of ER+ breast cancer therapy in all stages of the disease. In the metastatic disease setting, the use of ET agents is associated with clinical benefit in the majority of patients. Nonetheless, disease progression associated with a complexity of mechanisms of resistance remains a significant challenge [10].

ER, a protein encoded by the *ESR1* gene, is expressed in the majority of breast cancers. ER expression is one of the defining features in classifying tumor subtype and assigning therapeutic strategies in breast cancer. Translational and clinical research has established the fundamental role of ER and its hormonal ligands in normal mammary gland development and in the etiology and progression of breast cancer [11].

Estrogen hormones have genome-wide transcriptional activities that regulate the expression of a network of molecular pathways that are important in various physiological and pathological processes [12]. Functionally, the ER consists of two transcriptional activation domains: the *N*-terminal, ligand-independent activation function domain (AF-1), and the *C*-terminal, ligand-dependent AF-2 domain. The ligand-binding domain (LBD) resides in the *C*-terminal region, while the DNA-binding and hinge domains are positioned in the central core of the protein [2]. Estrogen binding triggers a number of events resulting in activation of ER and induces conformational changes in the LBD, allowing the estrogen-ER complex to bind to specific DNA sequences while interacting with corepressor and coactivator proteins to regulate the transcription of estrogen-responsive genes. Breast tumors undergo genomic evolution during therapy, with the development of new alterations that confer resistance to therapy. *ESR1* is known to undergo LBD mutations, gene amplification, or translocations that are potential mechanisms of resistance to ET [13–15].

Physiologically, estrogens promote a balanced activation of liganded and unliganded transcriptional functions of the ER. When ligand-dependent ER signaling is suppressed by either estrogen deficiency or dysfunction of the receptor, there is a strong upregulation of unliganded ER activation and subsequent resistance to endocrine therapies [16]. The absence of estrogen results in a compensatory increase in the activity of the AF-1 domain accompanied by a significant increase in the expression levels of both coding and noncoding RNA transcripts [17].

Despite the relatively high frequency of elevated *ESR1* copy numbers in breast tumors [18], the clinical relevance of *ESR1* gene amplification as a prognostic or predictive biomarker is not clear and requires further study [15]. However, mutations in the *ESR1* gene have been consistently recognized as an important mechanism of resistance to aromatase inhibitors (AIs), with a prevalence that ranges from 9 to 40%, usually described from liquid biopsies collected from patients mostly included in randomized clinical trials in developed countries [9, 19, 20].

**Figure 1:**
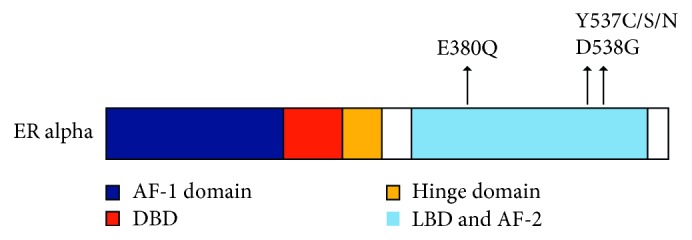
*ESR1* gene and most common mutations (reprinted with permission from Ma et al. [3]). A schematic diagram of *ESR1m* and their frequencies in ER+ advanced breast cancer after endocrine therapy. The structural domains of ER*α* are shown, including the transcription activation function 1 (AF-1) domain, the DNA-binding domain (DBD), the receptor dimerization and nuclear localization (hinge) domain, and the ligand-binding domain (LBD) and AF-2 domain.

*ESR1m* are most commonly missense mutations clustered in codons 537 and 538 of the LBD. Remarkably, the majority of *ESR1m* localize to just a few amino acids within or near the critical helix 12 region of the ER LBD, where they are likely to be single-allele mutations, as pictured in Figure 1 [3]. The most prevalent *ESR1* point mutations are Y537S and D538G, while several others have been identified at significantly lower frequencies. *ESR1m* have been consistently associated with inferior outcomes and is being evaluated as predictive biomarkers to help guide therapeutic decisions [21]. At the same time, the development of specific targeted therapies directed to *ESR1*-mutant clones is an appealing concept with interesting preclinical data already published and promising clinical work in progress [22, 23].

Our study reports that the prevalence of *ESR1m* in real-world patients with breast cancer in Brazil is similar to that described in the literature. This finding has implications related to the development of a line of research of mechanisms of ET resistance in the neoadjuvant setting as well as to the design and conduct the clinical trials evaluating new generation selective ER degraders (SERDs) in an *ESR1m*-enriched cohort of patients. Despite the low number of cases with mutation, our data show a significant association of visceral site of metastasis and *ESR1m*. Early studies reported *ESR1m* in tumor samples obtained from different sites, including visceral and nonvisceral metastasis, suggesting that these mutations do not display specific organotropism [24, 25]. Contrastingly, multivariable analyses based on liquid biopsies of patients from the PALOMA3 and SOFEA trials reported that the detection of *ESR1m* is associated with bone and visceral disease, suggesting that *ESR1m* are infrequently detected in locoregional recurrences [26, 27]. In our study, *ESR1* mutation was identified in locoregional and distant metastasis in a variety of visceral (lung, liver, pleura, and ovary) and nonvisceral sites (bone, chest wall, and lymph nodes) indicating that these mutations do not have organotropism and suggesting that this mechanism of ET resistance could be associated with more aggressive disease phenotypes that usually present with hepatic and pleuropulmonary metastasis.

The generation of real-world data is an issue with practical implications for global breast cancer research, and it remains a challenge, especially in low- to middle-income countries (LMIC). Translating clinical research achievements into global clinical practice is the clear objective. Clinical trials are designed and conducted in a controlled fashion with specific inclusion and exclusion criteria. Nonetheless, the confirmation of patients' characteristics and outcomes in a more general population remains an integral part of the process. Observational studies have demonstrated significant clinical and epidemiological differences among breast cancer patients compared to patients from developed countries, with a higher proportion of patients with locally-advanced tumors and young patients, especially among the population treated in the public health system [28].

Nevertheless, the potential differences in the molecular epidemiology of breast tumors in real-world patients from LMIC have not been adequately studied. The prevalence of biomarkers in breast cancer may vary in different regions of the world. A retrospective observational study with more than five thousand breast cancer patients demonstrated that the distribution of molecular subtypes of breast tumors differed according to geographic regions within Brazil and suggested that a variety of characteristics including socioeconomic and nutritional status as well as the proportion of African ancestry have to be considered to explain this heterogeneity [29]. It is important to understand the molecular characteristics of breast cancer in the Brazilian population in order to develop adequate public health programs and policies as well as to the development of therapeutic strategies and clinical trials. As an example, recently presented real-world data indicate a lower prevalence of PDL-1 expression in non-small-cell lung cancer patients in Brazil. The authors suggested that possible explanations for this discrepancy are inadequate sample handling, preanalytical issues, or epidemiology of the biomarker, all of which may have impacted the results of biomarkers outside clinical trials [30]. The unquestionable impact of breast cancer and the ongoing culture of globalization should be seen as opportunities to tackle critical global cancer research priorities, such as the development of research in LMIC, the encouragement of independent academic research, the improvement of access to clinical trials, and the development of international collaborations.

Our study has several limitations including its retrospective nature, relatively low sample size and the low number of *ERS1m* identified. Additionally, DNA extraction was unsuccessful in four samples of bone metastases, even though successful DNA extraction was achieved in the majority of bone samples (10 out of 14). We recognize that the detected prevalence of *ESR1m* can be underestimated given the fact that a PCR-based methodology was used and only specific mutations in the most commonly mutated codons were analyzed; therefore, cases with mutations in different codons of the *ESR1* gene potentially detectable with next-generation sequencing technologies were not identified [31, 32]. Another potentially important fact that might decrease the prevalence is that many patients in this cohort were treated with AIs in the adjuvant setting, whereas recent data suggest that *ESR1m* are probably more commonly associated with resistance to the AIs used in the metastatic disease setting [8].

This study is one of the first steps in a project of developing a comprehensive line of translational research in breast cancer through a collaboration of independent academic centers in Brazil. The publication of data of molecular biomarkers in real-world patients that are consistent with data from researches with patients treated in clinical trials is essential to allow validation of our methodology and to provide information for the development of translational and clinical research projects.

## 5. Conclusion

The prevalence of *ESR1m* in samples from Brazilian patients with metastatic ER+ breast cancer is similar to that described in patients included in clinical trials. A significant association between *ESR1m* and visceral site of metastasis was detected. *ESR1m* have potential clinical applications in breast cancer as a biomarker and a therapeutic target.

## Figures and Tables

**Table 1:** Association of *ESR1m* with the site of metastasis (*n* (%)).

	Visceral Metastasis	Nonvisceral metastasis	Total
*ESR1* mutation	8 (25.0%)	3 (6.7%)	11 (14.3%)
*ESR1* without mutation	24 (75.0%)	42 (93.3%)	66 (85.7%)
Total	32 (41.6%)	45 (58.4%)	77 (100.0%)

## Data Availability

The results reported in the article are publicly available and are included in an online file in Supplemental Materials.

## Supplementary Materials

Supplementary Materials Molecular biology analysis results. Each line represents a tumor sample, and the columns summarize information such as metastatic site, DNA quantification, the presence or absence of *ESR1m*, the mutant codon, and PCR cycle quantification value for each sample.
